# Supplementary material for: Splice-Junction-Based Mapping of Alternative Isoforms in the Human Proteome
Source: Cell Rep. Author manuscript; Available in PMC 2020 Jan 15. (PMC6961840; doi:10.1016/j.celrep.2019.11.026)
Supplement: 2 [file NIHMS1546469-supplement-2.zip › DF1/PXD010154/Spleen/Spleen_2_PCBP2_YSTGSDSASFPHTTPSMCLNPDLEGPPLELTK.pdf]

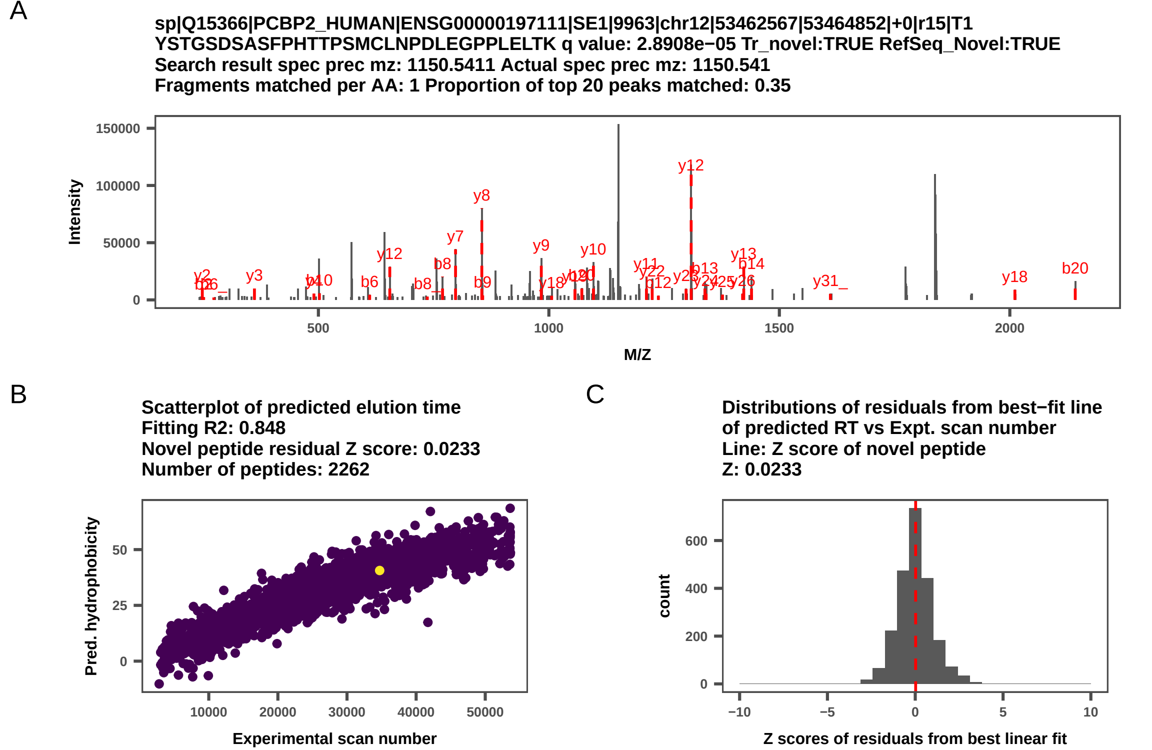
A
sp|Q15366|PCBP2_HUMAN|ENSG00000197111|SE1|9963|chr12|53462567|53464852|+0|r15|T1
YSTGSDSASFPHTTPSMCLNPDLEGPPLELTK q value: 2.8908e−05 Tr_novel:TRUE RefSeq_Novel:TRUE
Search result spec prec mz: 1150.5411 Actual spec prec mz: 1150.541
Fragments matched per AA: 1 Proportion of top 20 peaks matched: 0.35
Intensity
150000
100000
50000
0
500
1000
1500
2000
M/Z
y12
y8
y7
y9
y10
y13
b14
b20
y18
y31_
y3
y2
b6
b8
b9
b12
b13
B
Scatterplot of predicted elution time
Fitting R2: 0.848
Novel peptide residual Z score: 0.0233
Number of peptides: 2262
Pred. hydrophobicity
50
25
0
10000
20000
30000
40000
50000
Experimental scan number
C
Distributions of residuals from best−fit line
of predicted RT vs Expt. scan number
Line: Z score of novel peptide
Z: 0.0233
count
600
400
200
0
−10
−5
0
5
10
Z scores of residuals from best linear fit
